# Behavioral Complexity in Alzheimer’s Disease: A Diversity-Based Analysis of Neuropsychiatric Symptoms

**DOI:** 10.3390/brainsci16070659

**Published:** 2026-06-23

**Authors:** YoungSoon Yang, Yong Tae Kwak

**Affiliations:** 1 Department of Neurology, Soonchunhyang University College of Medicine, Cheonan Hospital, Cheonan 31151, Chungcheongnam-do, Republic of Korea; astro76@naver.com; 2 Department of Neurology, Hyoja Geriatric Hospital, Yongin 17089, Gyeonggi-do, Republic of Korea

**Keywords:** Alzheimer’s disease, neuropsychiatric symptoms, behavioral complexity, Shannon entropy, amyloid PET

## Abstract

**Highlights:**

**What are the main findings?**

• Behavioral complexity of neuropsychiatric symptoms in Alzheimer’s disease can be quantified using symptom count, normalized Shannon entropy, and a composite diversity-based index derived from 12 K-NPI domains.
• In amyloid PET–positive, psychotropic drug-naïve patients with probable Alzheimer’s disease, behavioral complexity was associated with greater dementia severity, and CDR remained independently associated with complexity even after adjustment for total neuropsychiatric burden.

**What are the implications of the main findings?**

• Neuropsychiatric symptoms in Alzheimer’s disease should not be evaluated only by total symptom burden; the distribution of symptoms across domains may provide additional clinically meaningful information.
• Entropy-based behavioral complexity may help identify patients with more diffuse multi-domain neuropsychiatric involvement, particularly when total symptom burden is still relatively low.

**Abstract:**

**Background and Objectives**: To quantify behavioral complexity in probable Alzheimer’s disease (AD), compare complexity phenotypes, and determine whether behavioral complexity provides clinically meaningful information beyond total neuropsychiatric burden. We also explored whether global amyloid extent and lobar amyloid topography added explanatory value. **Methods**: In this cross-sectional retrospective study, we analyzed 245 psychotropic drug-naïve patients with probable AD, positive ^18^F-FC119S amyloid positron emission tomography (PET), and complete neuropsychiatric, cognitive, functional, and regional PET data. Behavioral complexity was derived from 12 Korean Neuropsychiatric Inventory domains using symptom count, normalized Shannon entropy of the frequency × severity profile, and a composite index. Patients were classified into tertiles. Multivariable regression and burden-stratified analyses examined associations with cognition, dementia severity, function, and amyloid measures. **Results**: Higher behavioral complexity was associated with lower Korean Mini-Mental State Examination (K-MMSE) scores and higher Clinical Dementia Rating (CDR) and Global Deterioration Scale (GDS) stages. In multivariable analysis, higher CDR, higher GDS, and lower Barthel Index independently predicted greater complexity, whereas amyloid extent did not. After adjustment for total neuropsychiatric burden, higher CDR remained independently associated with the composite complexity index and normalized entropy, while amyloid extent remained non-significant. Complexity-related clinical differences were most evident in the lowest burden stratum and attenuated at higher burden levels. Regional amyloid analyses yielded only selective signals. **Conclusions**: Behavioral complexity is a clinically meaningful neuropsychiatric phenotype in AD. Although strongly related to total neuropsychiatric burden, it is not fully reducible to it, with its clearest independent association seen for global dementia severity, particularly at lower overall burden.

## 1. Introduction

Neuropsychiatric symptoms (NPS), often referred to as behavioral and psychological symptoms of dementia, are among the most clinically consequential manifestations of Alzheimer’s disease (AD). Although cognitive decline remains the core hallmark of AD, much of the day-to-day burden for patients and caregivers arises from agitation, psychosis, affective symptoms, and other behavioral disturbances. These symptoms are associated with poorer quality of life, greater caregiver distress, increased health-care utilization, and earlier institutionalization, and they complicate both pharmacological and non-pharmacological management [1,2,3].

A substantial literature has attempted to organize this heterogeneity. Factor-analytic and cluster-based studies of the Neuropsychiatric Inventory (NPI) have identified partially reproducible groupings such as psychosis, affective disturbance, apathy, hyperactivity, and vegetative change [4,5,6,7,8,9]. More recently, symptom-network approaches have examined how NPS co-occur and interact across patients [10]. These approaches have advanced understanding of behavioral structure across patient groups, but they are less suited to describing an individual-level feature often recognized in clinical practice: whether behavioral burden is relatively focal or broadly distributed across domains.

This distinction may be clinically important. Two patients may have similar overall NPI burden, yet one may present with a concentrated syndrome dominated by one or two domains, whereas another may exhibit multi-domain behavioral disturbance requiring broader management and greater caregiver support [1,2]. To address this gap, we focused on behavioral complexity as a patient-level expression of symptom diversity, defined here as the breadth and dispersion of neuropsychiatric involvement within an individual patient. The Korean Neuropsychiatric Inventory (K-NPI) provides a practical basis for operationalizing this construct because it captures both frequency and severity across 12 domains [11,12]. In the present study, breadth was represented by symptom count and dispersion by normalized Shannon entropy, and these were combined into a composite behavioral complexity index [13,14]. An important unresolved question is whether behavioral complexity is simply another way of describing overall symptom burden. Because NPS frequently co-occur across multiple NPI domains and can be organized into partially reproducible symptom groupings, patients with higher overall NPS burden may also show involvement of a greater number of domains, including delusions, hallucinations, agitation/aggression, depression, anxiety, euphoria, apathy, disinhibition, irritability, aberrant motor behavior, nighttime disturbance, and appetite/eating change [4,5,6,7,10]. This overlap creates an important methodological concern: any complexity metric based on symptom breadth or dispersion may partly reflect total symptom burden. For the construct to be clinically meaningful, it should provide information beyond total K-NPI burden.

We sought to determine whether behavioral complexity retained clinically meaningful associations beyond overall neuropsychiatric burden, even after adjustment for total burden. We also examined whether amyloid positron emission tomography (PET) added explanatory value within this framework. Although amyloid PET provides biological support for AD, the clinical expression of neuropsychiatric symptoms may depend more on downstream network dysfunction than on amyloid burden alone [3,15,16]. Regional amyloid topography may nevertheless be relevant, particularly in association cortices involved in large-scale behavioral networks [17,18,19]. Therefore, in an amyloid-positive probable AD cohort, we examined whether clinical severity, overall neuropsychiatric burden, amyloid extent, and lobar amyloid topography were associated with behavioral complexity.

## 2. Materials and Methods

### 2.1. Study Design and Participants

This retrospective observational study used an institutional database of psychotropic drug-naïve patients with clinically probable Alzheimer’s disease who consecutively attended the Soonchunhyang University Dementia Clinic between May 2020 and December 2025. Inclusion criteria were probable AD according to contemporary National Institute on Aging–Alzheimer’s Association (NIA-AA) principles, positive ^18^F-FC119S amyloid PET, no current psychotropic medication exposure, and complete K-NPI data [20,21]. For the present analyses, patients also required complete data for Korean Mini-Mental State Examination (K-MMSE), Clinical Dementia Rating (CDR), Global Deterioration Scale (GDS), Barthel Index, and regional amyloid PET topographic variables. The final analytic dataset comprised 245 patients, each represented once (Figure S1).

### 2.2. Clinical, Functional, and Neuropsychiatric Assessment

Cognition was summarized by the K-MMSE. Global dementia stage was assessed using the CDR and the GDS. Basic activities of daily living were summarized by the Barthel Index. The Barthel Index version used in this study ranges from 0 to 20, with higher scores indicating greater independence in basic activities of daily living. The K-NPI evaluates 12 domains: delusion, hallucination, aggression/agitation, depression, anxiety, euphoria, apathy, disinhibition, irritability, aberrant motor behavior, nighttime disturbance, and appetite/eating change [11,12]. For each K-NPI domain, frequency is rated from 1 to 4 and severity from 1 to 3 when a symptom is present; the domain-specific frequency × severity (FS) score therefore ranges from 1 to 12, while absent symptoms are scored as 0. Total K-NPI FS burden was calculated as the sum of the 12 domain-specific FS scores, yielding a possible range of 0 to 144. Higher total K-NPI FS burden indicates greater overall neuropsychiatric symptom burden. A domain was considered present when its FS score was greater than zero.

### 2.3. Behavioral Complexity Metrics

We conceptualized behavioral complexity as a patient-level diversity construct with two complementary components: breadth and dispersion. Breadth was represented by symptom count, defined as the number of K-NPI domains with FS > 0, with a possible range of 0 to 12. Dispersion was represented by normalized Shannon entropy. For each patient, the 12 domain-specific FS values were converted to relative weights by dividing each non-zero domain score by the patient’s total FS burden. Entropy was then computed as H = −Σ p_i log(p_i) across all domains with non-zero FS scores, where i indexes the 12 K-NPI domains and p_i denotes the proportion of the patient’s total FS burden contributed by domain i [13]. The entropy value was normalized by dividing by log(12), yielding a measure bounded between 0 and 1. Higher entropy indicates that behavioral burden is spread more evenly across multiple domains, whereas lower entropy indicates concentration in fewer domains. Because symptom count and normalized entropy capture related but non-identical aspects of behavioral complexity, we created a study-derived composite complexity index by converting symptom count and normalized entropy into cohort-based z scores and then summing the two z scores, following general principles for constructing composite variables from related measures on different scales [14]. Thus, higher composite index values indicate greater symptom diversity, characterized by both broader and more evenly distributed neuropsychiatric involvement. Patients were then divided into tertiles of the composite index and classified as having low, intermediate, or high behavioral complexity to facilitate interpretability and ensure approximately balanced group sizes.

### 2.4. PET Imaging and Derived Amyloid PET Variables

^18^F-FC119S PET/CT acquisition and interpretation followed the same institutional protocol as in our previous study [9,22,23]. ^18^F-FC119S was used because it is the routinely available clinical amyloid PET tracer at our institution and has demonstrated clinical utility for detecting cortical β-amyloid deposition [22,23]. All scans were acquired using a standardized static 3D protocol on a Siemens Biograph 6 TruePoint TrueV scanner. Participants received 370 ± 37 MBq of ^18^F-FC119S intravenously, and imaging was initiated 30 min after injection. Attenuation correction was performed using low-dose CT, and images were reconstructed with ordered-subset expectation maximization (4 iterations, 8 subsets). Global amyloid status was determined by consensus visual interpretation. Regional amyloid topography was derived from automated standardized uptake value ratio (SUVR)-based quantification using BTXBrain-Amyloid™ (version 1.3; Brightonix Imaging, Seoul, Republic of Korea), with whole cerebellar gray matter as the reference region. For the present analyses, six binary lobe-level indicators were defined: right frontal, left frontal, right temporal, left temporal, right parietal, and left parietal. Amyloid extent was calculated as the number of positive lobes across these six indicators.

### 2.5. Statistical Analysis

All analyses were performed at the patient level. Continuous variables are reported as mean ± standard deviation and categorical variables as counts with percentages. Across the three behavioral complexity tertiles, continuous variables were compared using one-way analysis of variance and categorical variables using Pearson’s chi-square tests. The base multivariable model used the composite complexity index as the dependent variable and included age, sex, education, K-MMSE, CDR, GDS, Barthel Index, and amyloid extent. To determine whether behavioral complexity provides information beyond total neuropsychiatric burden, we then fitted additional multivariable linear regression models with the composite complexity index, symptom count, and normalized entropy as dependent variables, each additionally adjusted for total K-NPI FS burden. To complement the regression approach, we performed FS-stratified analyses. Patients were grouped into four strata based on the empirical quartile distribution of total K-NPI FS burden. This approach was chosen to preserve reasonable subgroup sizes while allowing comparison of behavioral complexity across the full burden range without the instability of exact matching. Within each stratum, patients were classified as having lower versus higher behavioral complexity by a median split of the composite complexity index. To examine whether regional amyloid topography contributed additional information, we fitted a second set of multivariable linear regression models with the composite complexity index as the dependent variable and the same clinical covariates together with the six regional lobe indicators. All statistical tests were two-sided and *p*-values < 0.05 were considered statistically significant.

## 3. Results

### 3.1. Characteristics of the Patients

**Figure 1:**
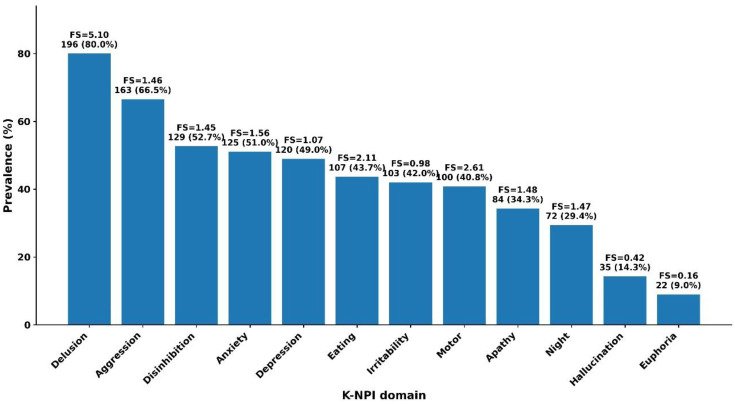
Prevalence of K-NPI domains with corresponding mean frequency × severity burden. Bars indicate the prevalence of each Korean Neuropsychiatric Inventory (K-NPI) domain, defined as frequency×severity (FS) > 0. Text labels show the corresponding mean FS score for each domain in the full cohort. Domains are ordered by prevalence.

The cohort comprised 245 patients with probable AD and positive ^18^F-FC119S amyloid PET. Mean age was 72.96 ± 7.84 years, 138 (56.3%) were women, and mean education was 10.19 ± 5.05 years. Mean K-MMSE was 18.92 ± 4.20, mean CDR was 1.41 ± 0.60, mean GDS was 4.20 ± 0.71, and mean Barthel Index was 17.58 ± 2.17. Across the full cohort, mean total K-NPI FS burden was 19.87 ± 13.67, mean symptom count was 5.13 ± 2.84, and mean normalized entropy was 0.50 ± 0.26 (Table 1). The most prevalent K-NPI domains were delusion (196/245, 80.0%), aggression (163/245, 66.5%), disinhibition (129/245, 52.7%), anxiety (125/245, 51.0%), and depression (120/245, 49.0%) (Figure 1). The prevalence of each individual K-NPI domain across behavioral complexity tertiles is presented in Table S1.

### 3.2. Behavioral Complexity Tertiles

Patients were divided into tertiles of the composite complexity index, yielding 83 low-complexity, 81 intermediate-complexity, and 81 high-complexity cases. As intended, the tertiles were strongly separated by symptom count (*p* < 0.001), total FS burden (*p* < 0.001), and entropy (*p* < 0.001). Mean symptom count increased from 2.04 ± 1.28 in the low-complexity group to 5.07 ± 0.82 in the intermediate group and 8.33 ± 1.42 in the high-complexity group. Total FS burden increased in parallel (8.20 ± 7.20, 20.49 ± 9.01, and 31.20 ± 12.81, respectively), and entropy rose from 0.20 ± 0.17 to 0.75 ± 0.08. Across tertiles, age, sex, and education did not differ significantly. By contrast, greater behavioral complexity was associated with worse cognition and more advanced disease stage, with lower K-MMSE and higher CDR and GDS scores across tertiles (Table 1). Barthel Index showed a numerical decline across tertiles but did not reach statistical significance on unadjusted ANOVA.

### 3.3. Amyloid Extent and Regional Topography

Mean amyloid extent in the full cohort was 1.67 ± 0.80 positive lobes (Table 1). The most frequently positive lobes were left temporal, left frontal, right temporal, and right frontal, whereas parietal positivity was uncommon (Figure S2). Amyloid extent differed modestly across behavioral complexity tertiles on unadjusted comparison, with a slightly higher mean extent in the high-complexity group than in the intermediate- or low-complexity groups. However, this gradient did not persist in multivariable analysis.

### 3.4. Multivariable Correlates of Behavioral Complexity

In the base full-cohort regression model, higher CDR (B = 0.583, 95% CI 0.113 to 1.052; *p* = 0.015), higher GDS stage (B = 0.639, 95% CI 0.249 to 1.029; *p* = 0.001), and lower Barthel Index (B = −0.109, 95% CI −0.217 to −0.001; *p* = 0.049) independently predicted higher behavioral complexity, whereas amyloid extent was not independently associated with complexity (B = 0.091, 95% CI −0.208 to 0.389; *p* = 0.551) (Table 2). Age, sex, education, and K-MMSE were not independent predictors after adjustment.

### 3.5. Behavioral Complexity Beyond Total Neuropsychiatric Burden

Because total K-NPI FS burden increased substantially across behavioral complexity tertiles, we next examined whether behavioral complexity retained clinical associations after accounting for overall symptom burden. In burden-adjusted models, total FS burden was strongly associated with all three behavioral complexity outcomes (Table 3). After adjustment for total FS, higher CDR remained independently associated with the composite complexity index (B = 0.344, 95% CI 0.022 to 0.665; *p* = 0.036) and normalized entropy (B = 0.052, 95% CI 0.008 to 0.096; *p* = 0.020). CDR showed a positive but non-significant association with symptom count (B = 0.396, *p* = 0.088). By contrast, GDS and Barthel Index were no longer independently associated with the composite complexity index after total FS was included. Amyloid extent remained non-significant in all burden-adjusted models. Total FS burden itself was a strong predictor of the composite complexity index (B = 0.106, *p* < 0.001), symptom count (B = 0.155, *p* < 0.001), and normalized entropy (B = 0.013, *p* < 0.001) (Table 3).

### 3.6. FS-Stratified Analyses Within Comparable Total Neuropsychiatric Burden

Within the lowest burden stratum (0–9), patients with higher complexity had lower K-MMSE (19.03 vs. 23.00, *p* = 0.003) and higher CDR (1.31 vs. 0.74, *p* < 0.001) than those with lower complexity. In this stratum, GDS, Barthel Index, and amyloid extent did not differ significantly. In the intermediate strata (10–19 and 20–28), differences between lower- and higher-complexity subgroups were small and not statistically significant across K-MMSE, CDR, GDS, Barthel Index, and amyloid extent. In the highest burden stratum (29–68), higher complexity was associated with a numerically lower Barthel Index (16.16 vs. 17.50), but this difference did not reach statistical significance (*p* = 0.067). As shown in Table 4, the clinical relevance of behavioral complexity within comparable total burden was most evident in the lower-burden range and became less consistent once total NPS burden reached moderate to high levels.

### 3.7. Exploratory Regional Topography Models

In exploratory regional models, left temporal positivity was inversely associated with the composite complexity index, whereas right temporal positivity appeared more closely related to total FS burden than to complexity itself. Right parietal positivity showed a borderline or selective relationship in some models (Table S2). Overall, regional amyloid topography yielded selective rather than dominant explanatory signals.

## 4. Discussion

This study examined neuropsychiatric symptoms in AD from the perspective of behavioral complexity, a patient-level diversity phenotype reflecting how broadly and evenly behavioral burden is distributed across domains. Four main conclusions emerged. First, behavioral complexity can be operationalized using symptom count and normalized Shannon entropy [13,14]. Second, greater complexity is associated with worse cognition and more advanced clinical severity in conventional analyses. Third, behavioral complexity is strongly related to total neuropsychiatric burden but is not completely reducible to it. Fourth, amyloid extent remains weakly informative, while regional amyloid topography provides only selective exploratory signals.

The most important implication of this analysis is that behavioral complexity is not equivalent to total NPI burden, but neither is it fully independent of it. In the burden-adjusted models, total K-NPI FS burden was by far the strongest predictor of the composite complexity index, symptom count, and entropy. This was expected, because patients with more overall symptom burden typically have more domains involved and greater dispersion across those domains. However, even after controlling for total FS, higher CDR remained independently associated with both the composite complexity index and normalized entropy. This suggests that behavioral complexity retains a residual link to global dementia severity beyond simple symptom load. At the same time, these findings also make the manuscript more conservative. Before adjustment for total K-NPI FS burden, higher GDS stage and lower Barthel Index were independently associated with greater behavioral complexity. After additional adjustment for total K-NPI FS burden, these associations were attenuated and no longer statistically significant. Thus, it would be too strong to claim that behavioral complexity represents a phenotype fully independent of disease severity. A more accurate interpretation is that behavioral complexity partly reflects more advanced clinical disease, but also captures the distributional pattern of neuropsychiatric involvement across domains. In this sense, behavioral complexity may be considered a distributional neuropsychiatric phenotype that overlaps with total symptom burden and disease severity, while retaining a residual clinical signal, most clearly for global dementia severity as indexed by CDR.

The FS-stratified analysis shown in Table 4 reinforces this more nuanced view. If behavioral complexity were uniformly informative across the entire burden spectrum, patients with higher complexity would be expected to show consistently worse clinical profiles within each FS stratum. That was not the case. The clearest differences emerged in the lowest burden stratum, where higher complexity was associated with lower K-MMSE and higher CDR. At moderate and high levels of total burden, however, the differences between lower- and higher-complexity subgroups were small and inconsistent. This pattern suggests that behavioral complexity may be most clinically informative early in the burden spectrum, when diffuse multi-domain symptom involvement may mark a less favorable phenotype despite relatively limited total NPS burden [24]. This interpretation is clinically plausible. Among patients with modest overall behavioral burden, a diffuse pattern may reflect broader network-level involvement than a focal syndrome of comparable total severity [25]. In contrast, once overall burden becomes high, total symptom load itself may dominate the clinical picture, leaving less additional information to be captured by how symptoms are distributed. Consistent with this interpretation, domain-level patterns in Table S1 provide a more concrete description of what higher behavioral complexity represents clinically. Higher behavioral complexity was accompanied by broader involvement across nearly all K-NPI domains. In the high-complexity tertile, frequently involved domains included delusion, aggression/agitation, anxiety, disinhibition, depression, irritability, aberrant motor behavior, and appetite/eating change. These findings suggest that higher complexity reflects broad multi-domain neuropsychiatric involvement rather than the dominance of a single symptom domain. However, because of the sample size of the present study, we did not formally decompose entropy into individual domain-specific contributions. This issue should be examined in future studies with larger datasets and additional biomarker information.

The PET findings remained limited. Amyloid extent did not independently predict complexity in the base model and did not emerge as important in the additional burden-adjusted analyses. This supports the view that lobar amyloid burden is unlikely to be the main determinant of whether a patient’s neuropsychiatric profile is relatively focal or behaviorally diffuse. That pattern is biologically plausible, because the expression of NPS in AD may depend more directly on downstream tau-related injury, synaptic dysfunction, large-scale network disconnection, and reduced reserve than on amyloid burden itself [15,16,21,26]. Exploratory regional analyses suggested that topography may still matter, but only selectively. Left temporal positivity remained inversely associated with the composite complexity index in the regional model, whereas right temporal positivity appeared more closely related to overall FS burden than to complexity itself. One possible interpretation is that some regional amyloid patterns may be linked more to the severity of a dominant symptom cluster, whereas behavioral complexity reflects how widely symptom burden is distributed across domains. However, these signals should be interpreted cautiously, as the regional PET variables were binary and anatomically coarse.

This study has several strengths. It was restricted to psychotropic drug-naïve, amyloid-positive probable AD, thereby reducing diagnostic and treatment-related heterogeneity [20,21]. It used the full 245-patient cohort and derived complexity from observed FS information across all K-NPI domains. Most importantly, it moved beyond the initial tertile comparison by explicitly testing whether behavioral complexity retained meaning after controlling for total burden and by examining complexity within comparable FS strata.

Several limitations should also be acknowledged. First, the study was cross-sectional, so behavioral complexity cannot be interpreted as a temporal progression marker. In addition, reliable information on disease duration was not consistently available in this retrospective registry. In Alzheimer’s disease, disease onset is difficult to determine precisely because it is usually estimated from patient or caregiver history and may vary depending on whether cognitive, functional, or behavioral symptoms are used as the reference point. Therefore, we could not determine whether disease duration differed across behavioral complexity tertiles or assess its potential influence on the association between behavioral complexity and clinical severity. Second, burden-adjusted models necessarily include variables that are related by construction, so the results should be interpreted as evidence of partial rather than absolute independence. Third, some FS strata contained modest subgroup sizes, limiting the precision of within-stratum comparisons. Fourth, PET analyses relied on binary lobar indicators. Fifth, no tau PET, structural MRI, or functional connectivity measures were available. Sixth, K-NPI ratings depend on caregiver report and remain vulnerable to measurement noise [11,12,15,16,17,18,19]. Finally, this study was based on a single retrospective cohort of amyloid PET–positive, psychotropic drug-naïve patients with probable AD. Therefore, replication in independent patient populations and other clinical settings will be necessary to establish the robustness and generalizability of the behavioral complexity model.

## 5. Conclusions

Behavioral complexity appears to represent a clinically meaningful neuropsychiatric phenotype in probable AD. It is strongly related to total neuropsychiatric burden, but is not entirely reducible to it. After adjustment for total burden, the most consistent independent correlate was global dementia severity as indexed by CDR, and within-stratum clinical differences were most evident in the lower-burden range. These findings support a nuanced view of behavioral complexity: it should not be treated as simply another name for total symptom severity, but neither should it be presented as broadly independent from overall neuropsychiatric burden.

## Figures and Tables

**Table 1:** Baseline demographic, clinical, behavioral, and PET characteristics of the overall cohort and according to behavioral complexity tertiles.

Variable	Overall	Low	Intermediate	High	*p*-Value
Number of patients	245	83	81	81	
Age, years	72.96 ± 7.84	72.45 ± 8.43	72.26 ± 7.12	74.17 ± 7.86	0.230
Female sex, n (%)	138 (56.3%)	46 (55.4%)	45 (55.6%)	47 (58.0%)	0.931
Education, years	10.19 ± 5.05	10.11 ± 5.52	10.27 ± 4.81	10.20 ± 4.84	0.980
K-MMSE	18.92 ± 4.20	20.28 ± 4.97 ^a^	18.81 ± 3.63 ^ab^	17.63 ± 3.43 ^b^	<0.001
CDR	1.41 ± 0.60	1.19 ± 0.64 ^a^	1.51 ± 0.56 ^b^	1.54 ± 0.54 ^b^	<0.001
GDS	4.20 ± 0.71	3.94 ± 0.80 ^a^	4.25 ± 0.70 ^b^	4.42 ± 0.52 ^b^	<0.001
Barthel Index	17.58 ± 2.17	17.92 ± 1.85	17.32 ± 2.34	17.48 ± 2.26	0.191
Symptom count	5.13 ± 2.84	2.04 ± 1.28 ^a^	5.07 ± 0.82 ^b^	8.33 ± 1.42 ^c^	<0.001
Total K-NPI FS	19.87 ± 13.67	8.20 ± 7.20 ^a^	20.49 ± 9.01 ^b^	31.20 ± 12.81 ^c^	<0.001
Normalized entropy	0.50 ± 0.26	0.20 ± 0.17 ^a^	0.55 ± 0.07 ^b^	0.75 ± 0.08 ^c^	<0.001
Amyloid extent (positive lobes)	1.67 ± 0.80	1.60 ± 0.81 ^ab^	1.56 ± 0.71 ^a^	1.86 ± 0.85 ^b^	0.029

Values are presented as mean ± standard deviation unless otherwise stated. *p*-values represent comparisons across the three behavioral complexity tertiles. Continuous variables were compared using one-way analysis of variance (ANOVA), and sex was compared using Pearson’s chi-square test. For continuous variables with significant overall group differences, post hoc pairwise comparisons were performed using Tukey’s test. Superscript letters indicate the results of post hoc comparisons; values that do not share the same letter are significantly different from each other, whereas values sharing the same letter are not significantly different. K-MMSE, Korean Mini-Mental State Examination; CDR, Clinical Dementia Rating; GDS, Global Deterioration Scale; K-NPI, Korean Neuropsychiatric Inventory; FS, frequency × severity.

**Table 2:** Multivariable correlates of the composite behavioral complexity index.

Predictor	B	SE	95% CI	*p*-Value
Age	0.015	0.016	−0.015 to 0.046	0.321
Female sex	0.225	0.277	−0.321 to 0.772	0.418
Education	−0.007	0.027	−0.059 to 0.046	0.795
K-MMSE	−0.036	0.037	−0.109 to 0.037	0.335
CDR	0.583	0.238	0.113 to 1.052	0.015
GDS	0.639	0.198	0.249 to 1.029	0.001
Barthel Index	−0.109	0.055	−0.217 to −0.001	0.049
Amyloid extent	0.091	0.151	−0.208 to 0.389	0.551

Multivariable linear regression model in the full cohort, with the composite behavioral complexity index as the dependent variable. Positive B values indicate greater behavioral complexity. Model R^2^ = 0.188; adjusted R^2^ = 0.160. K-MMSE, Korean Mini-Mental State Examination; CDR, Clinical Dementia Rating; GDS, Global Deterioration Scale.

**Table 3:** Multivariable linear regression models for behavioral complexity outcomes additionally adjusted for total K-NPI frequency × severity burden.

Variables	Composite Index B (SE)	*p*-Value	Symptom Count B (SE)	*p*-Value	Normalized Entropy B (SE)	*p*-Value
Age	0.002 (0.011)	0.855	0.003 (0.015)	0.855	0.000 (0.001)	0.865
Female sex	0.214 (0.190)	0.260	0.226 (0.269)	0.402	0.034 (0.026)	0.186
Education	−0.012 (0.018)	0.502	−0.014 (0.026)	0.594	−0.002 (0.003)	0.449
K-MMSE	−0.014 (0.026)	0.597	−0.021 (0.036)	0.555	−0.002 (0.003)	0.662
CDR	0.344 (0.163)	0.036	0.396 (0.231)	0.088	0.052 (0.022)	0.020
GDS	0.096 (0.138)	0.490	0.156 (0.196)	0.426	0.010 (0.019)	0.582
Barthel Index	0.041 (0.040)	0.304	0.050 (0.057)	0.382	0.006 (0.005)	0.268
Amyloid extent	0.078 (0.090)	0.385	0.097 (0.127)	0.444	0.011 (0.012)	0.362
Total K-NPI FS burden	0.106 (0.006)	<0.001	0.155 (0.009)	<0.001	0.013 (0.001)	<0.001

Multivariable linear regression models were fitted separately for the composite behavioral complexity index, symptom count, and normalized entropy. All models were adjusted for age, sex, education, K-MMSE, CDR, GDS, Barthel Index, amyloid extent, and total K-NPI frequency × severity (FS) burden. Total K-NPI FS burden was included as an additional covariate to assess whether associations were retained beyond overall neuropsychiatric burden. The composite complexity index was defined as the sum of z-standardized symptom count and normalized entropy.

**Table 4:** Clinical and functional comparison according to lower versus higher behavioral complexity within strata of comparable total K-NPI frequency × severity burden.

Total K-NPI FS Stratum	Complexity Subgroup	n	K-MMSE	CDR	GDS	Barthel Index	Amyloid Extent
0–9	Lower	34	23.00	0.74	3.56	18.24	0.77
	Higher	29	19.03 **	1.31 ***	3.76	17.55	0.79
10–19	Lower	32	18.00	1.58	4.31	17.62	0.84
	Higher	32	18.31	1.63	4.41	17.84	0.88
20–28	Lower	33	18.55	1.53	4.39	17.24	0.91
	Higher	32	17.53	1.52	4.50	18.09	0.91
29–68	Lower	28	18.43	1.57	4.39	17.50	1.00
	Higher	25	18.00	1.50	4.32	16.16	1.04

** *p* < 0.01; *** *p* < 0.001 for comparisons between lower- and higher-complexity groups within each FS stratum. Patientswere divided into quartile-based strata according to total K-NPI frequency × severity scores (0–9, 10–19, 20–28, and 29–68). Within each stratum, patients were further classified into lower- and higher-complexity groups using a median split of the composite complexity index. Only statistically significant between-group differences within each stratum are indicated in the table. K-NPI, Korean Neuropsychiatric Inventory; FS, frequency x severity; K-MMSE, Korean Mini-Mental State Examination; CDR, Clinical Dementia Rating; GDS, Global Deterioration Scale.

## Data Availability

The deidentified data and code used in this study are available from the corresponding author upon reasonable request for non-commercial research, subject to institutional review board approval and data use agreement.

## Supplementary Materials

The following supporting information can be downloaded at: https://www.mdpi.com/article/10.3390/brainsci16070659/s1, Figure S1: Flow diagram of patient selection for the analytic cohort; Figure S2: Frequency of regional amyloid PET positivity by lobe in the full cohort; Table S1: Prevalence of individual K-NPI domains by behavioral complexity tertile; Table S2: Exploratory multivariable linear regression models for behavioral complexity outcomes with regional amyloid topography.
