# Supplementary material for: Behavioral Complexity in Alzheimer’s Disease: A Diversity-Based Analysis of Neuropsychiatric Symptoms
Source: Brain Sci. 2026 Jun 23;16(7):659. doi: 10.3390/brainsci16070659 (PMC13406203; doi:10.3390/brainsci16070659)
Supplement: Supplementary file 1 [file brainsci-16-00659-s001.zip › Table S1.pdf]

Table S1. Prevalence of individual K-NPI domains by behavioral complexity tertile

Domain	Low (n=83)	Intermediate (n=81)	High (n=81)	p-Value
Delusion	48 (57.8%)	70 (86.4%)	78 (96.3%)	<0.001
Hallucination	0 (0.0%)	6 (7.4%)	29 (35.8%)	<0.001
Aggression	26 (31.3%)	62 (76.5%)	75 (92.6%)	<0.001
Depression	15 (18.1%)	42 (51.9%)	63 (77.8%)	<0.001
Anxiety	12 (14.5%)	43 (53.1%)	70 (86.4%)	<0.001
Euphoria	2 (2.4%)	3 (3.7%)	17 (21.0%)	<0.001
Apathy	7 (8.4%)	26 (32.1%)	51 (63.0%)	<0.001
Disinhibition	19 (22.9%)	44 (54.3%)	66 (81.5%)	<0.001
Irritability	10 (12.0%)	32 (39.5%)	61 (75.3%)	<0.001
Motor	8 (9.6%)	32 (39.5%)	60 (74.1%)	<0.001
Night	8 (9.6%)	19 (23.5%)	45 (55.6%)	<0.001
Eating	15 (18.1%)	32 (39.5%)	60 (74.1%)	<0.001

Domain prevalence was defined as frequency×severity (FS) > 0. p-values were derived from Pearson's chi-square tests across the three behavioral complexity tertiles.
