# Supplementary material for: Behavioral Complexity in Alzheimer’s Disease: A Diversity-Based Analysis of Neuropsychiatric Symptoms
Source: Brain Sci. 2026 Jun 23;16(7):659. doi: 10.3390/brainsci16070659 (PMC13406203; doi:10.3390/brainsci16070659)
Supplement: Supplementary file 1 [file brainsci-16-00659-s001.zip › Table S2.pdf]

Table S2. Exploratory multivariable linear regression models for behavioral complexity outcomes with regional amyloid topography

Regional predictor	Composite complexity index		Symptom count		Total K-NPI FS burden		Normalized entropy	
	B	p-value	B	p-value	B	p-value	B	p-value
**Right frontal**	-0.058	0.841	-0.072	0.864	1.904	0.343	-0.008	0.823
**Left frontal**	0.234	0.438	0.495	0.256	0.786	0.706	0.015	0.698
**Right temporal**	0.413	0.111	0.628	0.093	4.597	0.011	0.049	0.146
**Left temporal**	-0.668	0.021	-0.823	0.048	-1.873	0.346	-0.096	0.010
**Right parietal**	1.474	0.053	1.919	0.081	6.798	0.197	0.203	0.040
**Left parietal**	0.057	0.918	0.007	0.993	2.952	0.436	0.014	0.846

Multivariable linear regression models were fitted separately for the composite behavioral complexity index, symptom count, total K-NPI frequency×severity burden, and normalized entropy. All models were adjusted for age, sex, education, K-MMSE, CDR, GDS, and Barthel Index. Regional predictors were coded as six binary lobar amyloid PET indicators.
