# Supplementary figures and images for: Behavioral Complexity in Alzheimer’s Disease: A Diversity-Based Analysis of Neuropsychiatric Symptoms
Source: Brain Sci. 2026 Jun 23;16(7):659. doi: 10.3390/brainsci16070659 (PMC13406203; doi:10.3390/brainsci16070659)

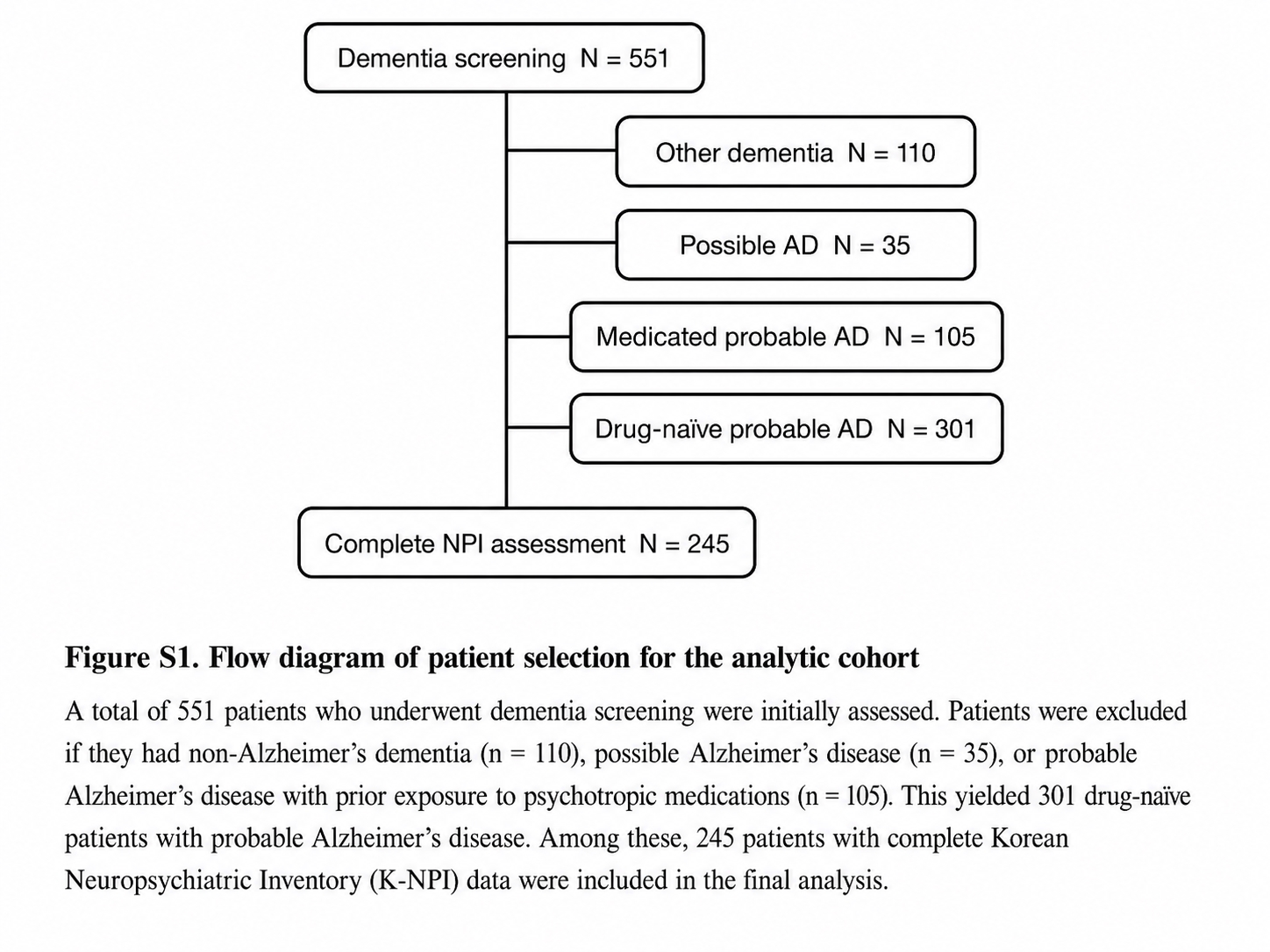

Supplement: Supplementary file 1 [file brainsci-16-00659-s001.zip › Figure S1.jpg]

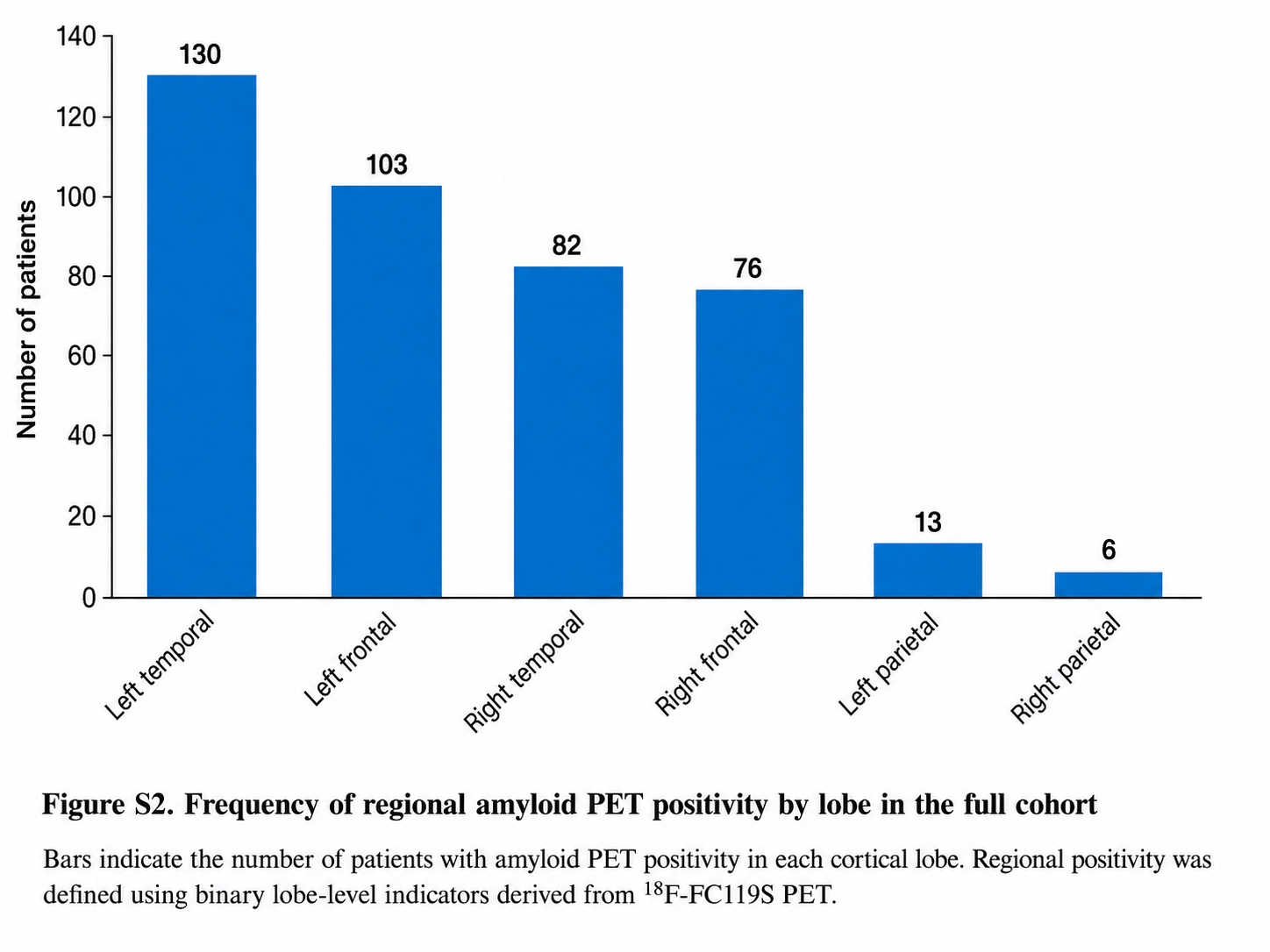

Supplement: Supplementary file 1 [file brainsci-16-00659-s001.zip › Figure S2.jpg]
